# Positive Selection and Centrality in the Yeast and Fly Protein-Protein Interaction Networks

**DOI:** 10.1155/2016/4658506

**Published:** 2016-03-28

**Authors:** Sandip Chakraborty, David Alvarez-Ponce

**Affiliations:** Department of Biology, University of Nevada, Reno, NV 89557, USA

## Abstract

Proteins within a molecular network are expected to be subject to different selective pressures depending on their relative hierarchical positions. However, it is not obvious what genes within a network should be more likely to evolve under positive selection. On one hand, only mutations at genes with a relatively high degree of control over adaptive phenotypes (such as those encoding highly connected proteins) are expected to be “seen” by natural selection. On the other hand, a high degree of pleiotropy at these genes is expected to hinder adaptation. Previous analyses of the human protein-protein interaction network have shown that genes under long-term, recurrent positive selection (as inferred from interspecific comparisons) tend to act at the periphery of the network. It is unknown, however, whether these trends apply to other organisms. Here, we show that long-term positive selection has preferentially targeted the periphery of the yeast interactome. Conversely, in flies, genes under positive selection encode significantly more connected and central proteins. These observations are not due to covariation of genes' adaptability and centrality with confounding factors. Therefore, the distribution of proteins encoded by genes under recurrent positive selection across protein-protein interaction networks varies from one species to another.

## 1. Introduction

Scientists have been fascinated for decades by the emergence and fixation of advantageous alleles by positive selection [1, 2]. Occasionally, a new mutation is beneficial or an existing mutation becomes beneficial due to a change in the environment. Under certain conditions, as individuals carrying such mutations have an increased fitness, these mutations can quickly spread through the population, leaving a characteristic footprint in the patterns of DNA variability [3, 4]. Certain genes are more likely than others to undergo positive selection, and understanding the reasons is essential to understand adaptation. The propensity of genes to undergo positive selection depends on the balance between the potential beneficial and deleterious effects of mutations at these genes [5]. On one hand, only genes whose variability has a considerable impact on the organism's fitness (i.e., genes with a high degree of control over advantageous traits) will be able to respond to natural selection [6, 7]. On the other hand, highly pleiotropic genes (at which mutations have a high likelihood of being deleterious) will less frequently respond to positive selection [1, 8–10]. Genes do not act in isolation; instead, they often function as parts of molecular pathways and networks. Both the importance and the degree of pleiotropy of genes are affected by their position within such networks, and therefore, a network framework may enable a better understanding of genes' different propensities to be targeted by positive selection.

Proteins within a molecular pathway or network have different relative impacts on the final output of the system (the phenotype, and ultimately fitness): alteration of certain key proteins profoundly impacts the behavior of the system, whereas alteration of other, less important proteins has only marginal effects [11]. The relative importance of proteins depends not only on their intrinsic properties (e.g., their kinetic properties), but also on the position that they occupy within the network. For instance, genes acting at the upstream part, or at bifurcating points of metabolic pathways, tend to have a great influence on metabolic flux [12–14], and proteins involved in many protein-protein interactions are often essential [15–17]. Proteins' degree of pleiotropy also depends on network position, with highly connected proteins, and those involved in a high number of pathways, being often highly pleiotropic. Therefore, genes acting at different parts of a network are expected to have different propensities to undergo positive selection, but it is often not obvious what parts of the network should be targeted more often by positive selection. Adaptive evolution is expected to target genes acting at key network positions (as less important proteins will rarely be seen by natural selection), particularly if the network is far from its adaptive optimum, but adaptation may be hindered by pleiotropy at these positions. Indeed, even though multiple studies have shown that genes' propensity to undergo adaptive evolution depends on their network position, clear rules have not emerged.

Population genetics studies based on a handful of well-defined metabolic and signaling pathways have so far suggested that positive selection often targets genes with relatively important pathway positions. For instance, positive selection acted on (i) genes encoding enzymes that act at bifurcating points of the* Drosophila melanogaster* pathways involved in glucose metabolism [18] and the human* N*-glycosylation pathway [19]; (ii) the gene encoding the first enzyme of the* Arabidopsis thaliana* glucosinolate pathway [13]; and (iii) genes encoding the most connected proteins in the human insulin/TOR pathway [20]. Beneficial mutations at genes acting at such key pathway positions may lead to rapid evolutionary change. Simulation analyses of evolving pathways suggest that, at the beginning of the adaptation process, when pathways are far away from their optimum, positive selection preferentially occurs at upstream genes, and at those acting at branch points; however, once pathways approach their optimum, upstream genes are highly constrained and downstream genes are the ones that undergo positive selection [12, 14].

In the last years, a considerable amount of genomic and functional data has accumulated, allowing evolutionary biologists to study the distribution of genes under positive selection in different kinds of large-scale networks. Genes under positive selection in honey bees, as inferred from the McDonald-Kreitman test [21], are lowly connected in the gene coexpression network [22]. In the* D. melanogaster* metabolic network, genes under positive selection, as inferred from the comparison of six* Drosophila* genomes, do not exhibit any particular network position; however, very few genes under positive selection were found in this study, which may have resulted in limited statistical power [23].

The relationship between genes' propensity to exhibit signatures of positive selection and the number of physical protein-protein interactions in which the encoded product is involved has only been addressed in humans, and contrasting trends have been observed depending on the evolutionary timescale considered. When recurring, long-term positive selection was inferred from comparison of the human and chimpanzee genomes [24], or from comparison of 10 mammalian genomes [16] (including 9 placentals and one marsupial, which diverged 157–170 million years ago [25]), using tests based on the nonsynonymous to synonymous divergence ratio (*ω* = *d*_*N*_/*d*_*S*_), positive selection was found to target preferentially genes acting at the periphery of the human protein-protein interaction network (i.e., genes encoding lowly connected proteins). Conversely, when recent selective sweeps were inferred from comparison of hundreds of human genomes, it was found that genes under positive selection were significantly more connected than genes with no signatures of positive selection [16, 26].

Are the trends observed thus far in the human protein-protein interaction network common to all organisms? Here, we characterize the distribution of genes under recurrent positive selection in the interactomes of* Saccharomyces cerevisiae* and* D. melanogaster*. For that purpose, we infer long-term positive selection events by comparing the genomes of five* Saccharomyces* and six* Drosophila* species. We find that, similar to what was previously observed in humans [16, 24], genes under positive selection act at the periphery of the* S. cerevisiae* protein-protein interaction network. Conversely, in* D. melanogaster*, genes under positive selection are significantly more connected than genes with no signatures of positive selection.

## 2. Materials and Methods

### 2.1. Tests of Positive Selection

For each* S. cerevisiae* gene, the longest encoded protein was selected for analysis, and orthologs were identified in another 4* Saccharomyces* genomes using a best reciprocal hit approach. Each* S. cerevisiae* longest protein was used as a query in BLASTP search (*E*-value cut-off: 10^−10^) against the proteomes of* S. paradoxus*,* S. mikatae*,* S. kudriavzevii*, and* S. bayanus*. The best hit in each proteome was used as a query in a second BLASTP search (*E*-value < 10^−10^) against the* S. cerevisiae* proteome. If the best hit identified in the second search was the original* S. cerevisiae* protein, then the encoding genes were considered to be orthologs. Only* S. cerevisiae* genes with identifiable orthologs in all four* Saccharomyces* species were used in our analyses. The same strategy was adopted to identify orthologs of all* D. melanogaster* genes in the genomes of* D. simulans*,* D. sechellia*,* D. yakuba*,* D. erecta*, and* D. ananassae*. We did not include more distant species in our analyses in order to (i) avoid saturation of synonymous sites; (ii) maintain a high number of analyzable genes (genes with orthologs in all considered species); and (iii) minimize problems resulting from alignment of highly divergent sequences.

Groups of orthologous protein sequences were aligned using ProbCons [27]. Given that gene annotations of nonmodel organisms are performed using automatic methods, which often produce imperfect gene models [28, 29], and that tests of positive selection are highly sensitive to such errors [30–33], we stringently filtered our protein sequence alignments using a three-step procedure (as in [16]). First, Gblocks version 0.91b [34] was used to eliminate nonalignable and poorly alignable regions. Second, a sliding window approach was used to identify alignment regions of 15 amino acids in which one of the sequences presented 10 or more singleton amino acids (amino acids that are unique to one sequence), and regions of 5 amino acids in which all were singletons in one of the species; such regions are unlikely to be correctly annotated. The original (unfiltered) sequence alignments were combined with the coding sequences (CDSs) and the results of the two first filtering steps to produce CDS alignments using an in-house pipeline. The resulting alignments were used in a test of positive selection using the M8 versus M7 test (see below). Third, for genes inferred to be under positive selection, CDS sequence alignments were visualized and erroneously annotated regions were manually removed using BioEdit version 7.2.5 [35] before rerunning the test of positive selection.

For each alignment, the presence of a set of codons with *ω* > 1 was inferred using the M8 versus M7 test [36]. The likelihood of alignment under the M8 and M7 models was estimated using the codeml program of the PAML package version 4.4d [37]. In order to alleviate the problem of local optima, all computations were repeated using three starting *ω* values (*ω* = 0.04, 0.4, and 4). Both models assume that codons' *ω* values follow a beta distribution, with values ranging from 0 to 1. The M8 model allows for an additional class of codons with *ω*_*s*_ > 1. The fit of both models was compared using a likelihood ratio test: twice the difference in the log-likelihood of both models [2Δ*ℓ* = 2 × (*ℓ*_M8_ − *ℓ*_M7_)] was assumed to follow a *χ* ^2^ distribution with two degrees of freedom [38]. Genes with a *P* value lower than 0.05 and *ω*_*s*_ higher than 1 were considered to be under positive selection. Analyses were repeated using a more stringent *P* value (*P* < 0.01 and *ω*_*s*_ > 1), and controlling the false discovery rate associated with multiple testing using the Benjamini and Hochberg approach (*q* < 0.1 and *ω*_*s*_ > 1) [39]. Unless stated otherwise, genes considered to be under positive selection throughout this study correspond to those with *P* < 0.05 and *ω*_*s*_ > 1.

### 2.2. Network Data and Analyses

Protein-protein interaction data for* S. cerevisiae* and* D. melanogaster* were obtained from the BioGRID database version 3.4.129 [40]. This database contains only experimentally determined interactions. Only physical nonredundant interactions between proteins from the same organism were used in our analyses. Additional analyses were conducted using interaction data from the STRING database version 10 [41]. This database contains data from both experimentally determined and computationally predicted (based on coexpression, phylogenetic profiles, etc.) interactions. Only interactions with a confidence score ≥40% were used in our analyses.

For each protein and network, degree was computed as the number of other proteins with which the protein interacts, betweenness was computed as the number of shortest paths among other proteins that pass through the protein [42], and closeness was computed as one divided by the average distance (number of steps) between the protein and all other proteins. Betweenness and closeness computations were conducted using Pajek version 4.05 [43].

### 2.3. Protein Abundance and Gene Expression Data

Protein abundance data for* S. cerevisiae* and for the whole body of* D. melanogaster* adults was obtained from the PaxDB database version 4 [44]. Messenger RNA abundance data for* S. cerevisiae* was obtained from [45]. Messenger RNA abundance data for the whole* D. melanogaster* adult and 16 adult nonredundant tissues/organs were obtained from the FlyAtlas database [46]. Probes were mapped to genes using the Affymetrix annotation file “Drosophila 2” version 35. Probes matching multiple genes were not used in our analyses. For genes matching multiple probes, the probe with the highest mRNA abundance in the whole fly was used. The expression breadth of each* D. melanogaster* gene was computed as the number of tissues/organs in which the gene is expressed. The considered tissues were brain, head, eye, thoracicoabdominal ganglion, salivary gland, crop, midgut, tubule, hindgut, heart, fat body, ovary, testis, male accessory glands, virgin spermatheca, and carcass. A gene was considered to be expressed in a tissue/organ if the database reported presence in at least 3 out of the 4 biological replicates.

### 2.4. Number of Publications

For each* S. cerevisiae* gene, the number of publications in which it is referred was obtained from the* Saccharomyces* Genome Database [47]. The number of publications related to each* D. melanogaster* gene was obtained from FlyBase [48]. These data were obtained in February 2016.

## 3. Results

### 3.1. Positive Selection Acted Preferentially at the Periphery of the Yeast Protein-Protein Interaction Network

We identified the orthologs of all* S. cerevisiae* genes in the genomes of another four* Saccharomyces* genomes. A total of 2071* S. cerevisiae* genes had identifiable orthologs in all four genomes. Sequence alignments were filtered using highly stringent criteria, and the filtered alignments were used in a maximum likelihood test of positive selection [36]. A total of 91 genes exhibited signatures of positive selection according to our initial criteria (*P* < 0.05 and *ω*_*s*_ > 1). This number is moderately higher than that resulting from a scan based on three* Saccharomyces* genomes [49].

We reconstructed the yeast protein-protein interaction network from the experimentally determined physical protein-protein interactions recorded in the BioGRID database [40]. The network contained a total of 5864 nonredundant proteins and 81,040 nonredundant interactions (Table S1 in Supplementary Material available online at http://dx.doi.org/10.1155/2016/4658506). Out of the 5864 genes encoding the proteins represented in the network, 89 exhibited signatures of positive selection, 1956 did not exhibit signatures of positive selection, and in the remaining genes the test could not be performed, as orthologs were not identified in all yeast species.

**Figure 1:**
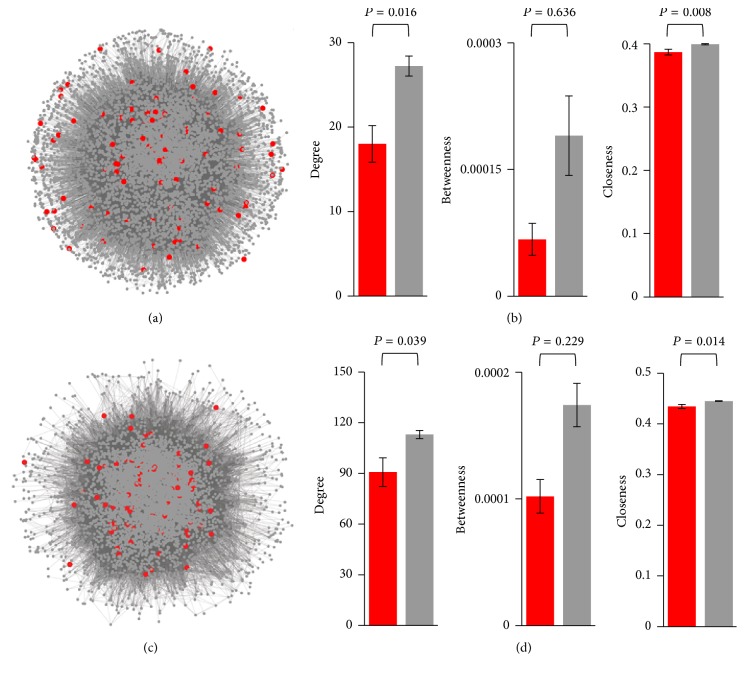
Distribution of proteins encoded by genes under positive selection in the* S. cerevisiae* protein-protein interaction network. (a) BioGRID network. (b) Average network centrality metrics calculated from the BioGRID network. (c) STRING network. (d) Average network centrality metrics calculated from the STRING network. In panels (a) and (c), proteins encoded by genes under positive selection are represented in red, and the rest of the proteins are represented in gray. In panels (b) and (d), genes under positive selection are represented in red, and genes with no signatures of positive selection are represented in gray. Error bars correspond to the standard error of the mean. Genes were considered to be under positive selection if they exhibited *P* < 0.05 and *ω*_*s*_ > 1. For analyses based on more stringent criteria, see Tables S3 and S4. *P* values represented in the figure correspond to the Mann-Whitney *U* test.

Genes with signatures of positive selection encode proteins that exhibit a significantly lower number of interactions (average for genes under positive selection: 18.01; average for genes with no signatures of positive selection: 27.23; Mann-Whitney *U* test, *P* = 0.016) and a significantly lower closeness centrality (mean for genes under positive selection: 0.387; mean for genes without signatures of positive selection: 0.400; *P* = 0.008). Genes under positive selection also exhibit a substantially but not significantly lower betweenness centrality (average for genes under positive selection: 6.73 × 10^−5^; average for genes with no signatures of positive selection: 1.90 × 10^−4^; *P* = 0.636) (Figures 1(a) and 1(b); Table S2). When a more stringent *P* value cut-off was applied in our tests of positive selection (*P* < 0.01), only 31 network genes were considered to be under positive selection. When the results of the tests of positive selection were corrected for multiple testing (*q* < 0.1), only 5 of these genes remained significant. Both gene sets encoded proteins with a substantially lower degree and betweenness, but differences were not significant, probably due to limited statistical power resulting from the small sample sizes (Tables S3 and S4).

We repeated our network analyses using a denser network obtained from the data recorded in the STRING database, which contains not only experimentally determined but also computationally predicted protein and gene interactions [41] (Table S1). Similar results were obtained: proteins encoded by genes under positive selection exhibit a significantly lower degree and closeness and a substantially, but not significantly, lower betweenness (Figures 1(c) and 1(d); Table S2). No significant differences were observed when more stringent criteria were used in the tests of positive selection (*P* < 0.01 or *q* < 0.1; Tables S3 and S4).

**Figure 2:**
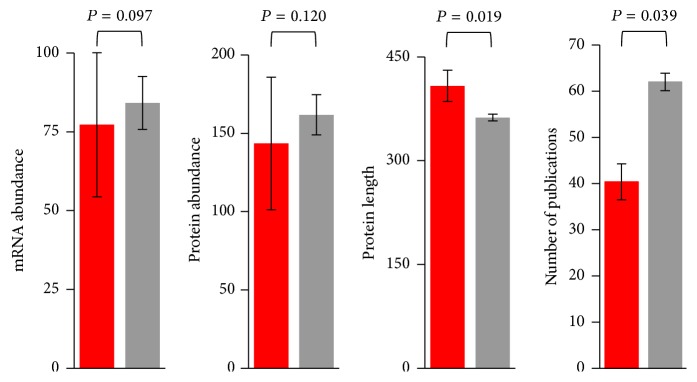
Differences in mRNA abundance, protein abundance, protein length, and number of publications between positively selected genes (in red) and genes without signatures of positive selection (in gray) in* S. cerevisiae*. Genes were considered to be under positive selection if they exhibited *P* < 0.05 and *ω*_*s*_ > 1. *P* values represented in the figure correspond to the Mann-Whitney *U* test.

We next considered whether our observations might be due to covariation of both gene adaptability and network centrality with different potentially confounding factors, rather than to a direct link between adaptability and centrality. Previous results in yeasts and other organisms have shown that central genes tend to be highly expressed [50–58], and previous works in humans have shown that highly expressed genes are unlikely to undergo adaptive evolution [24, 59]. Combined, these observations raise the possibility that our observations could merely be a byproduct of the distribution of expression levels in the network. We found a positive correlation between degree and both mRNA abundance (BioGRID network: Spearman's rank correlation coefficient, *ρ* = 0.294, *P* = 9.6 × 10^−37^; STRING network: *ρ* = 0.320, *P* = 7.1 × 10^−44^) and protein abundance (BioGRID network: *ρ* = 0.452, *P* = 2.1 × 10^−103^; STRING network: *ρ* = 0.441, *P* = 5.8 × 10^−99^). However, we found no differences between the expression levels and protein abundances of genes under positive selection and genes with no signatures of positive selection in yeast (Figure 2), which allowed us to discard these factors as the reason underlying our observations.

Also consistent with previous results in yeasts [60], we observed a positive correlation between proteins' length and number of protein-protein interactions (BioGRID network: *ρ* = 0.076, *P* = 6.3 × 10^−4^; STRING network: *ρ* = 0.078, *P* = 4.1 × 10^−4^). In line with previous results in mammals [16], we found that yeast genes under positive selection encode significantly longer proteins than those with no signatures of positive selection (Figure 2), which is consistent with the power of the test depending on the number of codons analyzed [61]. Combined, these observations indicate that our observation that genes under positive selection tend to encode peripheral genes cannot be due to covariation with protein length.

Interactomic datasets are known to be subjected to a number of biases [62]. In particular, such datasets tend to include a disproportionally high number of interactions involving proteins that have been studied in great detail (e.g., because of their particular importance or interest), as more resources have been devoted to study them. Indeed, we observed that protein degrees positively correlate with the number of publications mentioning the proteins (BioGRID network: *ρ* = 0.651, *P* < 10^−6^; STRING network: *ρ* = 0.553, *P* < 10^−6^) and that genes under positive selection tend to be mentioned in a lower number of publications (*P* = 0.039; Figure 2). Nonetheless, this bias is unlikely to explain our observations: a partial correlation analysis shows that, in the STRING network, 2Δ*ℓ* correlates with degree, even when controlling for the number of publications (*ρ* = −0.066, *P* = 0.003). In addition, when we used a subnetwork of the BioGRID network containing only the interactions determined by high-throughput techniques (which are expected to be less prone to this kind of bias) the difference between the degree of proteins encoded by genes under positive selection (mean = 15.31, median = 9) and the degree of the proteins encoded by genes with no signatures of positive selection (mean = 22.18, median = 11) remains substantially and marginally significantly different (*P* = 0.058).

Another known problem of currently available interactomes is their high rate of false positives [63–65]. To alleviate this problem, we generated two highly stringent subnetworks of our BioGRID and STRING networks. The first subnetwork was generated by considering only those protein-protein interactions determined by low-throughput techniques (which are expected to produce more reliable results than high-throughput techniques). In this case, the difference between the degree of proteins encoded by genes under positive selection (mean = 7.62, median = 5) and the degree of proteins encoded by genes with no signatures of positive selection (mean = 9.69, median = 5) remained substantial; however, the differences were not statistically significant (*P* = 0.808), probably owing to the reduced statistical power resulting from filtering the network. The second subnetwork was obtained by considering only the interactions described in the STRING database with a confidence score ≥50%. The degrees of proteins encoded by genes under positive selection were significantly lower (genes under positive selection: mean = 65.87, median = 43; genes with no signatures of positive selection: mean = 80.93, median = 54; *P* = 0.047). When an even more stringent cut-off was applied (score ≥90%), the differences were even more marked, but nonsignificant (positively selected: mean = 22.05, median = 9.5; non-positively selected: mean = 27.92, median = 12; *P* = 0.177), probably due to reduced statistical power.

### 3.2. Positive Selection Acted Preferentially at the Center of the Fly Protein-Protein Interaction Network

We performed a scan of positive selection using the genomes of six* Drosophila* species. Orthologs in all species were found for 10,340* D. melanogaster* genes, and signatures of positive selection were detected in 533 of these genes using a *P* value threshold of 0.05. This number is smaller than those resulting from previous scans of positive selection in* Drosophila* [31, 66], as expected from the fact that we applied highly stringent criteria, including manual inspection and editing of the alignments in which positive selection was detected (Section 2). The protein-protein interaction network, constructed from the data available at the BioGRID database [40], consisted of 7968 nonredundant proteins and 36,589 nonredundant interactions (Table S1). Out of the 7968 genes whose encoded products are represented in the network, positive selection was inferred in 350, no signatures of positive selection were found in 6171, and the positive selection test could not be performed on the rest because orthologs were not present in some of the genomes.

**Figure 3:**
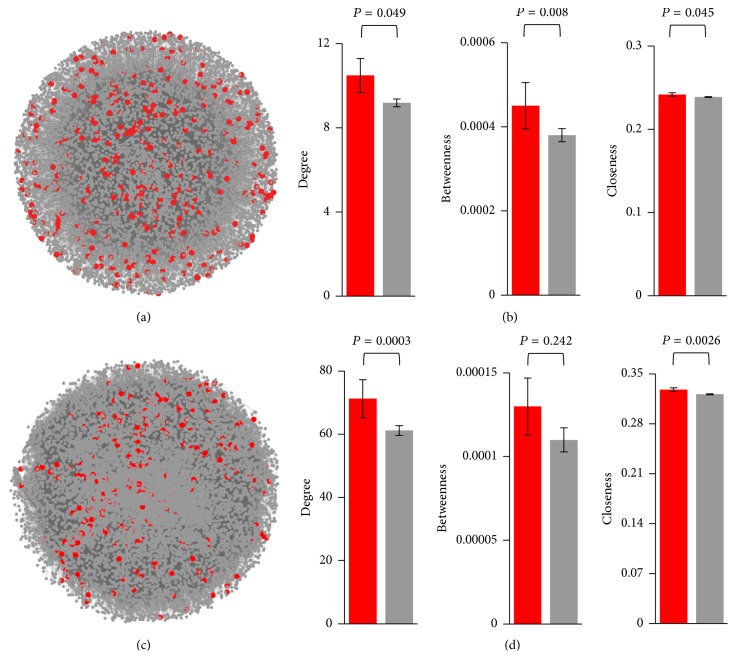
Distribution of proteins encoded by genes under positive selection in the* D. melanogaster* protein-protein interaction network. (a) BioGRID network. (b) Average network centrality metrics calculated from the BioGRID network. (c) STRING network. (d) Average network centrality metrics calculated from the STRING network. In panels (a) and (c), proteins encoded by genes under positive selection are represented in red, and the rest of the proteins are represented in gray. In panels (b) and (d), genes under positive selection are represented in red, and genes with no signatures of positive selection are represented in gray. Error bars correspond to the standard error of the mean. Genes were considered to be under positive selection if they exhibited *P* < 0.05 and *ω*_*s*_ > 1. For analyses based on more stringent criteria, see Tables S6 and S7. *P* values represented in the figure correspond to the Mann-Whitney *U* test.

Remarkably, we found that, contrary to what was found in yeast (Figure 1; Table S2) and humans [16, 24], genes under positive selection encoded proteins with a significantly higher degree (average for genes under positive selection: 10.49; average for genes with no signatures of positive selection: 9.19; *P* = 0.049), betweenness (average for genes under positive selection: 4.5 × 10^−4^; average for genes with no signatures of positive selection: 3.8 × 10^−4^; *P* = 0.008), and closeness (average for genes under positive selection: 0.242; average for genes with no signatures of positive selection: 0.239; *P* = 0.045) (Figures 3(a) and 3(b), Table S5). Similar results were obtained when a more stringent *P* value cut-off was used (*P* < 0.01), with the only exception that the differences are not significant, albeit substantial, for betweenness (Table S6). When the results of the positive selection test were corrected for multiple testing by controlling the false discovery rate (*q* < 0.1), only 50 network genes retained signatures of positive selection. These genes exhibit a higher degree, betweenness, and closeness, even though the differences are not statistically significant, probably due to a reduced statistical power due to the small genes under positive selection (Table S7). Similar results were also obtained when the network was assembled from the contents of the STRING database [41]; in this case, the differences are statistically significant for degree and closeness when a *P* value cut-off of *P* < 0.05 was used to detect positive selection (Figures 3(c) and 3(d), Table S5), and for degree, betweenness, and closeness when the more stringent cut-off of *P* < 0.01 was used (Table S6) or when correction for multiple testing was applied (*q* < 0.1; Table S7).

**Figure 4:**
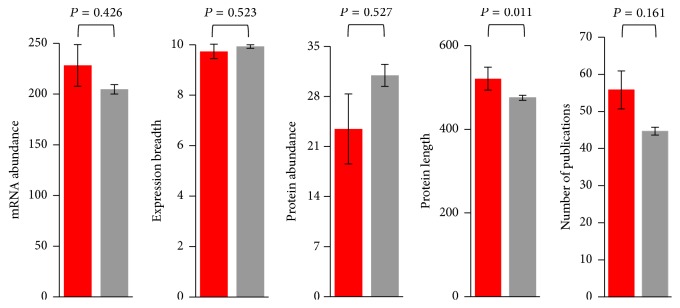
Differences in mRNA abundance, expression breadth, protein abundance, protein length, and number of publications between positively selected genes (in red) and genes without signatures of positive selection (in gray) in* D. melanogaster*. Genes were considered to be under positive selection if they exhibited *P* < 0.05 and *ω*_*s*_ > 1. *P* values represented in the figure correspond to the Mann-Whitney *U* test.

**Figure 5:**
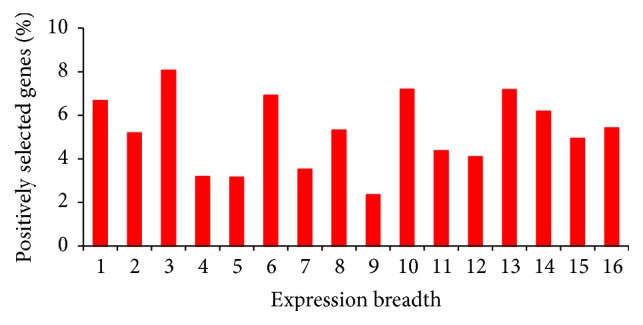
Percent of genes under positive selection among genes with different expression breadths in* D. melanogaster*. Genes were considered to be under positive selection if they exhibited *P* < 0.05 and *ω*_*s*_ > 1.

Consistent with previous results in* Drosophila* and other organisms [50–58], protein degree positively correlates with mRNA abundance (BioGRID network: *ρ* = 0.097, *P* = 1.1 × 10^−81^; STRING network: *ρ* = 0.338, *P* = 8.2 × 10^−114^). We also found degree to positively correlate with protein abundance (BioGRID network: *ρ* = 0.120, *P* = 4.3 × 10^−10^; STRING network: *ρ* = 0.288, *P* = 1.8 × 10^−83^) and with expression breadth (BioGRID network: *ρ* = 0.179, *P* = 2.2 × 10^−47^; STRING network: *ρ* = 0.036, *P* = 0.017). However, none of these parameters significantly differs between genes under positive selection and genes with no signatures of positive selection (Figure 4). In addition, genes with different expression breadths do not differ in their propensity to exhibit signatures of positive selection (correlation between expression breadth and the fraction of genes under positive selection: *ρ* = 0.018, *P* = 0.948; Figure 5). These observations allow us to discard the possibility that the observed higher centrality of genes under positive selection (Figure 3; Tables S5–S7) could be due to covariation of adaptability and centrality with these expression parameters. Similar to what is observed in yeast (Figure 2) and humans [16], genes under positive selection tend to encode long proteins in* Drosophila* (Figure 4). However, protein length does not correlate with number of interactions (BioGRID network: *ρ* = 0.028, *P* = 0.133; STRING network: *ρ* = 0.009, *P* = 0.555), indicating that our observations are not due to covariation with protein length either.

Protein degrees were found to positively correlate with the number of publications mentioning each protein (BioGRID network: *ρ* = 0.249, *P* < 10^−6^; STRING network: *ρ* = 0.251, *P* < 10^−6^). However, three lines of evidence demonstrate that this has not biased our results. First, the average number of publications does not significantly differ between genes under positive selection and genes with no signatures of positive selection (*P* = 0.161; Figure 4). Second, we repeated our analyses using the* D. melanogaster* protein-protein interaction network generated by Giot et al. [67]. This network is the result of a large-scale experiment in which virtually every possible interaction was tested, and therefore it is expected to be unbiased. Similar to our analyses on the entire BioGRID network, we observed that proteins encoded by genes under positive selection exhibited a higher degree (genes under positive selection: mean = 6.50, median = 3; genes with no signatures of positive selection: mean = 5.81, median = 3; *P* = 0.224). Third, when we repeated our analyses on a subnetwork containing only those interactions determined by high-throughput techniques, we observed significant differences between proteins encoded by genes under positive selection and those encoded by proteins with no signatures of positive selection (positively selected: mean = 10.42, median = 4; non-positively selected: mean = 8.97, median = 4; *P* = 0.046).

Finally, we repeated our analyses on two highly stringent subnetworks of our BioGRID and STRING networks. When we considered only those protein-protein interactions determined by low-throughput experiments, proteins encoded by genes under positive selection remained substantially more central; however, the differences were not statistically significant, probably due to reduced statistical power resulting from filtering our network (positively selected: mean = 2.91, median = 1; non-positively selected: mean = 2.56, median = 1; *P* = 0.708). When we considered only the interactions described in the STRING database with a confidence score ≥50% or ≥60%, proteins encoded by genes under positive selection remained significantly more central (*P* = 2.3 × 10^−3^ and 8.1 × 10^−3^, resp.). When only interactions with a score ≥90% were considered, the differences were even more marked, but nonsignificant (genes under positive selection: mean = 15.15, median = 4; genes with no signatures of positive selection: mean = 12.89, median = 4; *P* = 0.756), probably due to reduced statistical power.

## 4. Discussion

We have performed two scans of positive selection by comparing the genomes of five* Saccharomyces* and six* Drosophila* species and investigated the position of the proteins encoded by genes under positive selection in the protein-protein interaction networks of* S. cerevisiae* and* D. melanogaster*. Consistent with previous results in humans [16, 24], we found that genes under positive selection encode significantly less connected proteins in the interactome of* S. cerevisiae*. However, the opposite was observed in* Drosophila*: proteins encoded by genes under positive selection are significantly more connected than those encoded by genes with no signatures of positive selection. These observations were not due to covariation of network centrality and positive selection with protein abundance, expression level, protein length, or, in the case of* Drosophila*, expression breadth. Equivalent results were obtained when considering betweenness and closeness (two descriptors of the global position of proteins within the network, which take into account not only their direct interactors, but rather their potential role in connecting different parts of the network [42, 68]). Equivalent results were also obtained by analyzing the STRING database [41], which contains both experimentally determined and computationally predicted gene interactions.

Our observations imply that interactome position has an impact on the propensity of genes to undergo adaptive evolution. In other words, genes under positive selection do not distribute randomly in protein-protein interaction networks. Previous studies in humans have suggested that the relationship between network centrality and the propensity of genes to undergo positive selection depends on the timescale considered: genes that underwent positive selection recurrently during long evolutionary times, as revealed from comparison of the genomes of different species, act at the periphery of the human interactome [16, 24], whereas genes that underwent positive selection recently, as inferred from comparison of human genomes, encode highly interactive proteins [16, 26]. Our study shows that the distribution of genes under positive selection in the protein-protein interaction network also varies from one species to another.

The test of positive selection that we used [36] can detect adaptation events that affected the protein sequence in a recurring manner during long evolutionary periods (e.g., as a result of an arms race dynamics [69]), and not adaptation events in the regulatory region. Therefore, our study focused on the adaptation at the protein sequence level (likely protein function), rather than at the regulatory level. This was also the case of the study by Kim et al. [24], and the interspecific analysis conducted by Luisi et al. [16]. In contrast, studies in which positive selection was inferred from the SNP frequency spectrum, estimated from comparison of DNA sequences of alleles of the same species (e.g., the population genomics studies conducted by Luisi et al. [16] and by Qian et al. [26]), may have captured recent adaptation events, at both the regulatory and the protein sequence level.

Genes within a network have different hierarchical positions and, therefore, a different relative potential to affect adaptive phenotypes. In the context of protein-protein interaction networks, centrality is a proxy for this relative importance. Mutations affecting proteins involved in a high number of protein-protein interactions, or those with a high global centrality, are expected to have a high influence on network dynamics, and to have highly pleiotropic effects (indeed, highly pleiotropic genes tend to encode highly interactive proteins [70]). Consistently, genes encoding central proteins are often essential [15–17] and highly constrained by purifying selection [15, 16, 50, 71, 72].

At least two opposing forces may determine the direction of the relationship between genes' adaptability and network centrality. On one hand, beneficial mutations at genes encoding the “key” proteins of the network (e.g., those involved in a high number of protein-protein interactions) are likely to have a great impact on phenotypes and fitness, whereas beneficial mutations at genes encoding less important proteins (those whose variability does not impact much the final output of the network) will rarely be seen by natural selection. This is expected to result in positive selection targeting preferentially genes acting at the center of the network. On the other hand, pleiotropy is thought to constrain the adaptation of protein sequences. Mutations at genes involved in many biological processes, or in many interactions, are expected to affect a high number of phenotypes, thus making it unlikely that such genes can experience drastic changes at the protein sequence level [1, 8–10]. In addition, purifying selection may rapidly remove most nonsynonymous mutations from these genes, further hindering adaptation at the protein level. This is expected to result in positive selection acting preferentially at the network periphery. Nonetheless, compensatory mutations may promote recurring adaptation events at highly pleiotropic proteins. Adaptation of one aspect of the function of a protein may have negative side effects on other aspects of its function, which can be ameliorated or restored by subsequent adaptation events [73, 74].

It is possible that the balance between both forces is different in yeasts and* Drosophila* and that it is also different when considering the long-term evolutionary history of mammals* versus* the recent evolutionary history of humans. However, it is not obvious why the balance might be different in different organisms and/or timescales. One factor affecting the relative importance of both forces may be the point of the adaptive landscape in which a population is. Adaptive walks often proceed by an initial period of fixation of large-effect adaptive mutations, followed by fixation of mutations of smaller effects [1, 75, 76]. Populations that are poorly adapted to the environment (e.g., due to an environmental change) might undergo big adaptive leaps by fixing mutations at highly central genes. Conversely, populations that are near their adaptive optimum may undergo adaptation preferentially at the network periphery. Consistent with this model of diminishing returns, simulation of the adaptation of hypothetical, randomly generated metabolic pathways has shown that the first steps of adaptation (when pathways are far away from their optimal function) take place through positive selection acting at upstream genes, and those acting at branch points (the ones with a higher degree of control over the pathway flux), whereas at the end of the simulations (when pathways are near the optimum) pathways are fine-tuned by positive selection at downstream genes (which have a smaller influence on flux) [12, 14]. It is unclear, however, why the* Drosophila* network would be far away from its optimal functioning compared to the yeast and mammalian networks.

Effective population size (*N*_*e*_) may be another key modulator of the centrality of genes under positive selection. In organisms with large *N*_*e*_, the efficacy of natural selection is high, and even mutations with small selection coefficients will be fixed or removed by natural selection. This is expected to result in genes under positive selection at both the center and the periphery of the network. Conversely, in organisms with small *N*_*e*_, genetic drift can outpower natural selection, and only mutations with large effects are expected to be fixed/removed by natural selection [6], which is expected to result in positive selection mostly at the center of the network. Nonetheless, this is unlikely to explain the different trends observed in yeasts,* Drosophila*, and humans, as* D. melanogaster* is thought to have *N*_*e*_ higher than humans and lower than yeast (e.g., see [77–80]).

Another potentially important consideration is the so-called “cost of complexity.” Large-size mutations will more often be disruptive in complex organisms (those with many characters) than in simple ones [1, 81]. This may promote adaptation at the periphery of the networks of complex organisms. However, it is again unlikely that this factor has caused the observed differences between* Drosophila* and yeasts and mammals, as* Drosophila* exhibits an intermediate complexity between yeasts and mammals [82, 83]. The cost of complexity might be partially reduced by network modularity, as it may significantly reduce the pleiotropic effects of adaptive mutations by containing genes in small areas of influence [84, 85]. Nonetheless, there is no reason to think that the* Drosophila* interactome is more modular than those of yeast and human [86, 87], and* Drosophila* genes under positive selection exhibit high betweenness and closeness centralities, which is not compatible with their being confined in modules.

The five* Saccharomyces* species used in our analysis (all belonging to the* Saccharomyces sensu stricto* complex) diverged 10–20 million years ago [88]. The six* Drosophila* species analyzed in this study (all belonging to the* melanogaster* group) diverged ~30 million years ago [89]. Kim et al. [24] inferred positive selection from comparison of the human and chimpanzee genomes, which diverged ~6 million years ago [90, 91], and the 10 mammalian genomes studied by Luisi et al. [16] diverged 157–170 million years ago [25]. Therefore, the divergence time considered in our scan of positive selection in* Drosophila* falls within the range of the divergence times for the species used in the scans for the other taxa, indicating that the peculiar distribution of genes under positive selection within the* Drosophila* network is not due to divergence times.

Therefore, it is unclear why the distribution of genes under recurrent positive selection is different in the* Drosophila* interactome and in the human and yeast interactomes. In any case, our observations imply that even though network position is a key factor determining genes' propensity to undergo positive selection, the relationship between both factors is complex and lineage-specific.

## Supplementary Material

Table S1 shows the number of proteins and interactions of the networks used in the study.
Table S2 compares the network centralities of positively selected genes and genes with no signatures of positive selection in *S. cerevisiae*. In this table, genes were considered to be under positive selection if *P* < 0.05 and *ω_s_* > 1.
Table S3 compares the network centralities of positively selected genes and genes with no signatures of positive selection in *S. cerevisiae*. In this table, genes were considered to be under positive selection if *P* < 0.01 and *ω_s_* > 1.
Table S4 compares the network centralities of positively selected genes and genes with no signatures of positive selection in *S. cerevisiae*. In this table, genes were considered to be under positive selection if *q* < 0.1 and *ω_s_* > 1.
Table S5 compares the network centralities of positively selected genes and genes with no signatures of positive selection in *D. melanogaster*. In this table, genes were considered to be under positive selection if *P* < 0.05 and *ω_s_* > 1.
Table S6 compares the network centralities of positively selected genes and genes with no signatures of positive selection in *D. melanogaster*. In this table, genes were considered to be under positive selection if *P* < 0.01 and *ω_s_* > 1.
Table S7 compares the network centralities of positively selected genes and genes with no signatures of positive selection in *D. melanogaster*. In this table, genes were considered to be under positive selection if *q* < 0.1 and *ω_s_* > 1.
