# Supplementary material for: Positive Selection and Centrality in the Yeast and Fly Protein-Protein Interaction Networks
Source: Biomed Res Int. 2016 Mar 28;2016:4658506. doi: 10.1155/2016/4658506 (PMC4826914; doi:10.1155/2016/4658506)
Supplement: Supplementary file 1 — Table S1 shows the number of proteins and interactions of the networks used in the study. Table S2 compares the network centralities of positively selected genes and genes with no signatures of positive selection in S. cerevisiae. In this table, genes were considered to be under positive selection if P < 0.05 and ωs > 1. Table S3 compares the network centralities of positively selected genes and genes with no signatures of positive selection in S. cerevisiae. In this table, genes were considered to be under positive selection if P < 0.01 and ωs > 1. Table S4 compares the network centralities of positively selected genes and genes with no signatures of positive selection in S. cerevisiae. In this table, genes were considered to be under positive selection if q < 0.1 and ωs > 1. Table S5 compares the network centralities of positively selected genes and genes with no signatures of positive selection in D. melanogaster. In this table, genes were considered to be under positive selection if P < 0.05 and ωs > 1. Table S6 compares the network centralities of positively selected genes and genes with no signatures of positive selection in D. melanogaster. In this table, genes were considered to be under positive selection if P < 0.01 and ωs > 1. Table S7 compares the network centralities of positively selected genes and genes with no signatures of positive selection in D. melanogaster. In this table, genes were considered to be under positive selection if q < 0.1 and ωs > 1. [file 4658506.f1.pdf]

SUPPLEMENTARY MATERIAL

Positive selection and centrality in the yeast and fly protein–protein interaction networks

Sandip Chakraborty and David Alvarez-Ponce

Department of Biology, University of Nevada, Reno, Nevada 89557, US.

Table S1. Summary statistics of the networks used in the study

Organism	Database	Number of proteins	Number of interactions
S. cerevisiae	BioGRID	5864	81,040
	STRING (score ≥ 40%)	6303	332,039
D. melanogaster	BioGRID	7968	36,589
	STRING (score ≥ 40%)	12,223	454,083

Table S2. Network centralities of positively selected genes (P < 0.05) and genes with no signatures of positive selection in *S. cerevisiae*

		BioGRID			STRING		
		**Selected genes (n = 89)**	**Other genes (n = 1956)**	***P*-value**	**Selected genes (n = 91)**	**Other genes (n = 1976)**	***P*-value**
Degree	Mean	18.01	27.23	0.016*	90.76	113.04	0.039*
	Median	13	14		68	78	
Betweenness	Mean	6.73×10^{-5}	1.90×10^{-4}	0.636	1.02×10^{-4}	1.74×10^{-4}	0.229
	Median	1.21×10^{-5}	2.34×10^{-5}		6.36×10^{-5}	7.40×10^{-5}	
Closeness	Mean	0.3869	0.3995	0.008**	0.4342	0.4449	0.014*
	Median	0.3943	0.4045		0.4485	0.4518	

P-values correspond to the Mann–Whitney *U* test. *, $P < 0.05$; **, $P < 0.01$.

Table S3. Network centralities of positively selected genes (*P* < 0.01) and genes with no signatures of positive selection in *S. cerevisiae*

		BioGRID			STRING		
		Selected genes (n = 31)	Other genes (n = 2014)	*P*-value	Selected genes (n = 33)	Other genes (n = 2034)	*P*-value
Degree	Mean	20.45	26.93	0.810	102.73	112.21	0.792
	Median	15	14		76	77	
Betweennes	Mean	8.6×10^{-5}	1.9×10^{-4}	0.540	1.2×10^{-4}	1.7×10^{-4}	0.475
	Median	1.7×10^{-5}	2.3×10^{-5}		6.3×10^{-5}	7.3×10^{-5}	
Closeness	Mean	0.4066	0.3989	0.350	0.4451	0.4444	0.718
	Median	0.4115	0.4037		0.4531	0.4515	

P-values correspond to the Mann–Whitney *U* test.

Table S4. Network centralities of positively selected genes ($q < 0.1$) and genes with no signatures of positive selection in *S. cerevisiae*

		BioGRID			STRING		
		Selected genes ($n = 5$)	Other genes ($n = 2040$)	*P*-value	Selected genes ($n = 5$)	Other genes ($n = 2062$)	*P*-value
Degree	Mean	15.60	26.86	0.516	71.40	112.16	0.608
	Median	9	14		76	77	
Betweennes	Mean	3.8×10^{-5}	1.90×10^{-4}	0.764	2.1×10^{-4}	1.7×10^{-4}	0.782
	Median	1.0×10^{-5}	2.29×10^{-5}		1.1×10^{-4}	7.3×10^{-5}	
Closeness	Mean	0.4141	0.3990	0.369	0.4474	0.4444	0.818
	Median	0.4190	0.4037		0.4497	0.4516	

P-values correspond to the Mann–Whitney *U* test.

Table S5. Network centralities of positively selected genes (*P* < 0.05) and genes with no signatures of positive selection in *D. melanogaster*

		BioGRID			STRING		
		**Selected genes (*n* = 350)**	**Other genes (*n* = 6171)**	***P*-value**	**Selected genes (*n* = 247)**	**Other genes (*n* = 4167)**	***P*-value**
Degree	Mean	10.49	9.19	0.049*	71.23	61.17	0.0004***
	Median	4	4		35	23	
Betweenness	Mean	4.5×10^{-4}	3.8×10^{-4}	0.008**	1.3×10^{-4}	1.1×10^{-4}	0.242
	Median	5.0×10^{-5}	3.0×10^{-5}		4.0×10^{-5}	2.0×10^{-5}	
Closeness	Mean	0.2418	0.2387	0.045*	0.3280	0.3214	0.0075**
	Median	0.2439	0.2406		0.3312	0.3243	

P-values correspond to the Mann–Whitney *U* test. *, *P* < 0.05; **, *P* < 0.01; ***, *P* < 0.001.

Table S6. Network centralities of positively selected genes (*P* < 0.01) and genes with no signatures of positive selection in *D. melanogaster*

		BioGRID			STRING		
		Selected genes (*n* = 139)	Other genes (*n* = 6373)	*P*-value	Selected genes (*n* = 95)	Other genes (*n* = 4310)	*P*-value
Degree	Mean	11.27	9.21	0.018*	80.57	61.32	2.7×10^{-5}***
	Median	5	4		51	23	
Betweenness	Mean	5.1×10^{-4}	3.8×10^{-4}	0.053	1.7×10^{-4}	1.0×10^{-4}	0.022*
	Median	6.2×10^{-5}	3.2×10^{-5}		6.2×10^{-5}	2.0×10^{-5}	
Closeness	Mean	0.2456	0.2387	0.006**	0.3304	0.3215	0.006**
	Median	0.2473	0.2406		0.3359	0.3243	

P-values correspond to the Mann–Whitney *U* test. *, *P* < 0.05; **, *P* < 0.01; ***, *P* < 0.001.

Table S7. Network centralities of positively selected genes (q < 0.1) and genes with no signatures of positive selection in *D. melanogaster*

		BioGRID			STRING		
		**Selected genes (n = 50)**	**Other genes (n = 6462)**	***P*-value**	**Selected genes (n = 33)**	**Other genes (n = 4167)**	***P*-value**
Degree	Mean	10.16	9.25	0.542	102.15	61.42	4.2×10^{-4}***
	Median	4	4		72	23	
Betweennes	Mean	4.3×10^{-4}	3.9×10^{-4}	0.717	2.5×10^{-4}	1.1×10^{-4}	0.002**
	Median	3.1×10^{-5}	3.2×10^{-5}		9.4×10^{-5}	2.1×10^{-5}	
Closeness	Mean	0.24612	0.23883	0.154	0.33877	0.32160	0.009**
	Median	0.24267	0.24070		0.34371	0.32445	

P-values correspond to the Mann–Whitney *U* test. *, $P < 0.05$; **, $P < 0.01$; ***, $P < 0.001$.
